# Application of a non-parametric non-mixture cure rate model for analyzing the survival of patients with colorectal cancer in Iran

**DOI:** 10.4178/epih.e2018045

**Published:** 2018-09-17

**Authors:** Mehdi Azizmohammad Looha, Mohamad Amin Pourhoseingholi, Maryam Nasserinejad, Hadis Najafimehr, Mohammad Reza Zali

**Affiliations:** 1 Department of Biostatistics, Faculty of Paramedical Sciences, Shahid Beheshti University of Medical Science, Tehran, Iran; 2 Gastroenterology and Liver Diseases Research Center, Research Institute for Gastroenterology and Liver Diseases, Shahid Beheshti University of Medical Sciences, Tehran, Iran; 3 Basic and Molecular Epidemiology of Gastrointestinal Disorders Research Center, Research Institute for Gastroenterology and Liver Diseases, Shahid Beheshti University of Medical Sciences, Tehran, Iran

**Keywords:** Colorectal cancer, Survival analysis, Non-mixture cure rate models, Body mass index, Iran

## Abstract

**OBJECTIVES:**

Colorectal cancer (CRC) patients are considered to have been cured when the mortality rate of individuals with the disease returns to the same level as expected in the general population. This study aimed to assess the impact of various risk factors on the cure fraction of CRC patients using a real dataset of Iranian CRC patients with a non-mixture non-parametric cure model.

**METHODS:**

This study was conducted on the medical records of 512 patients who were definitively diagnosed with CRC at Taleghani Hospital, Tehran, Iran from 2001 to 2007. A non-mixture non-parametric cure rate model was applied to the data after using stepwise selection to identify the risk factors of CRC.

**RESULTS:**

For non-cured cases, the mean survival time was 1,243.83 days (95% confidence interval [CI], 1,174.65 to 1,313.00) and the median survival time was 1,493.00 days (95% CI, 1,398.67 to 1,587.33). The 1- and 3-year survival rates were 92.9% (95% CI, 91.0 to 95.0) and 73.4% (95% CI, 68.0 to 79.0), respectively. Pathologic stage T1 of the primary tumor (estimate=0.58; p=0.013), a poorly differentiated tumor (estimate=1.17; p<0.001), a body mass index (BMI) between 18.6 and 24.9 kg/m^2^ (estimate=−0.60; p=0.04), and a BMI between 25.0 and 29.9 kg/m^2^ (estimate=−1.43; p<0.001) had significant impacts on the cure fraction of CRC in the multivariate analysis. The proportion of cured patients was 64.1% (95% CI, 56.7 to 72.4).

**CONCLUSIONS:**

This study found that the pathologic stage of the primary tumor, tumor grade, and BMI were potential risk factors that had an impact on the cure fraction. A non-mixture non-parametric cure rate model provides a flexible framework for accurately determining the impact of risk factors on the long-term survival of patients with CRC.

## INTRODUCTION

Cancer is one of the leading causes of morbidity and mortality worldwide, and it is the second leading cause of death globally [1]. One of the most important cancers is colorectal cancer (CRC), which has various incidence and mortality rates throughout the world [2]. The distribution of the CRC burden varies according to the human development index (HDI), with over half of the deaths occurring in high-HDI countries [3]. In many medium- to high-HDI countries, particularly in South America, Eastern Europe, and Asia, both CRC incidence and mortality have increased. In contrast, in many countries with the highest HDIs, such as the US, Australia, New Zealand, and several Western European countries, CRC incidence and mortality have either remained steady or decreased [4].

CRC is the third most commonly diagnosed malignancy, and it is usually related to changes in many different genes. A small proportion of CRC cases are due to hereditary genetic mutations [3,5]. More generally, CRC is a multifactorial disease, with risk factors including low physical activity, a high-fat diet, low intake of vegetables and fruit, alcohol consumption, high body mass index (BMI), a family history, and tobacco smoking [6]. In Iran, CRC is the fourth leading cause of cancer. Because of lifestyle and diet, the incidence of CRC is relatively high, and has risen in the last decades. It is between 7.0 and 8.0 per 100,000 persons per year in both genders in Iran [7-9]. The annual CRC mortality rate per 100,000 persons has increased in the last decade, and CRC is now the cancer with the fifth-highest standardized mortality rate in Iran [10,11]. In addition, the prevalence of CRC is roughly 6.0 to 7.9 per 100,000 persons in Iran [12]. Medical researchers have achieved significant progress in the diagnosis and treatment of CRC, and early detection of CRC by colonoscopy can increase patients’ survival time. In addition, high-quality surgery, adjuvant radiotherapy, and chemotherapy play an important role in achieving good outcomes [13]. Patients in whom CRC was detected by surveillance have been found to survive longer than patients in whom CRC was diagnosed on the basis of symptoms [14].

Survival analysis is a statistical method that involves analyzing the time to some event of interest. In classical survival analysis, cases ultimately experience the event of interest. However, it often happens that patients with cancer can be long-term survivors of their disease, and such patients are considered to be “cured.” In such circumstances, the Kaplan-Meier plot has a long and stable plateau, with heavy censoring at the extreme right of the plot, and therefore the classical survival model as a proper survival function is not a useful tool to analyze such cancer survival data. Various parametric and non-parametric approaches have been considered to model the cure fraction, which corresponds to the proportion of patients who are cured of their disease [15-17]. There are 2 major classes of cure models; the newest one includes non-mixture cure rate models, also known as promotion time cure models and bounded cumulative hazard models. This type of cure model has a proportional hazard structure. For a statistical analysis of survival data in the presence of a cure fraction, some distribution can be used to fit the baseline survival function for susceptible individuals. However, sometimes no distributions are fitted to the susceptible survival function and the analysis is done in a non-parametric manner. In such a situation, there is no problem finding the best distribution for the baseline survival function of susceptible individuals [18-21].

We aimed to investigate the effect of the clinical, pathological, and biological characteristics of patients with CRC on their survival using a non-parametric non-mixture cure model.

## MATERIALS AND METHODS

This study was conducted on the medical records of 512 patients who were pathologically diagnosed with CRC. These CRC patients were referred to Taleghani Medical and Training Hospital, Tehran, Iran, between 2001 and 2007. The patients’ information was extracted from their records from the hospital and health centers. The patients or patients’ family members were contacted by phone to confirm whether the patients were still alive. Deaths due to CRC were regarded as failure, and the survival time was calculated as the time interval between the diagnosis of CRC and death. Patients who survived to the end of the study were right-censored.

Age at diagnosis, gender, marital status, tobacco, alcohol history, family history, abdominal pain, weight loss, BMI, tumor grade, tumor size, pathologic regional lymph node staging, pathologic distant metastasis staging, and pathologic primary tumor staging were included in the current study as risk factors.

### Statistical analysis

Descriptive statistics, including frequency and percentage, were used to express the categorical characteristics of CRC patients. The log-rank test was used to assess the impact of each variable on the survival time. Forward stepwise variable selection was applied to investigate the best subset of characteristics in which the best fit of the proportional hazard model could be obtained. The variance inflation factor (VIF) was assessed to determine the presence of multicollinearity between the characteristics. Variables affected by multicollinearity were eliminated from the study, and the best subset was eventually selected.

A non-parametric non-mixture cure model was implemented and the log (-log) link function was used for the cure fraction in the current data. The backfitting procedure was used to maximize the non-parametric likelihood of the model and to estimate the model parameters of the promotion time cure model. Because the log (-log) link function was used, positive parameter estimation led to a smaller probability of the cure fraction and negative parameter estimation led to a larger probability of the cure fraction. Kaplan-Meier plots and the log-rank test were employed for evaluating the survival curve according to each patient characteristic. Survival data analysis was performed using the R packages miCoPTCM version 1.0 (https://cran.r-project.org/) and stepwise with 0.05 level of significance.

## RESULTS

A total of 512 patients were included in the study, comprising 309 (60.3%) men and 203 (39.6%) women, as shown in Table 1. For the non-cured cases, the mean survival time was 1,243.83 days (95% confidence interval [CI], 1,174.65 to 1,313.00) and the median survival time was 1,493.00 days (95% CI, 1,398.67 to 1587.33). The 1- and 3-year survival rates were 92.9% (95% CI, 91.0 to 95.0) and 73.4% (95% CI, 68.0 to 79.0), respectively. Of all patients, 88 died (17.8% of the men and 16.3% of the women).

**Figure 1.**
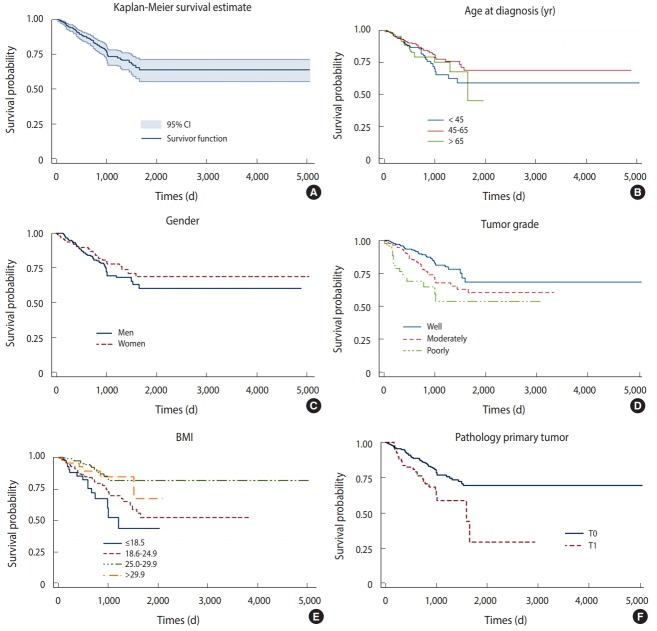
Survival probability of CRC patients (A) overall, (B) age at diagnosis, (C) gender, (D) tumor grade, (E) body mass index (BMI), and (F) pathologic stage of the primary tumor. CI, confidence interval.

Age at diagnosis, BMI, tumor grade, gender, and pathologic stage of the primary tumor were selected based on forward stepwise and VIF analysis. The proportional hazard assumption in the Cox regression model was rejected (p<0.001), meaning that using a Cox proportional model was no longer appropriate. The Kaplan-Meier survival plot showed a stable plateau at about 64.1% (95% CI, 57.0 to 72.0) that was reached at 1,654 days. Women had a slightly longer survival estimate than men throughout almost the entire study period. The survival curve by age at diagnosis indicated lower survival in the oldest age group. Patients aged between 45 and 65 at diagnosis had the highest survival estimates. CRC patients with poorly- and well-differentiated tumors had the lowest and highest survival probabilities, respectively, according to a plot analyzing patients by tumor grade. The BMI graph indicated that with increasing BMI, survival probability increased. The curve analyzing patients by pathologic stage of the primary tumor indicated that those with T0 tumors had a longer survival estimate than those with T1 tumors (Figure 1).

Initially, all covariates were included in the model with no interactions among them, and then, each variable was examined in the model. We found no significant association in the univariate or multivariate method between the cure probability of individuals and their age at diagnosis or gender; however, tumor grade, pathologic stage of the primary tumor, and BMI had a significant impact on the cure fraction of CRC patients. In the current data, patients older than 65 years at diagnosis had a higher probability of being cured than the patients in the reference category, but this probability was smaller for patients aged between 45 and 65 at diagnosis. Patients with BMIs of all non-underweight categories (18.6-24.9, 25.0-29.9, and >29.9 kg/m^2^) had a cure probability lower than that of patients in the reference category. A higher cure probability was seen in those with well differentiated tumors as a reference category than in those with moderately and poorly differentiated tumors. The probability of being cured was higher in women than in men. Furthermore, patients with a pathologic T1 primary tumor had a higher probability of cure than those with T0 primary tumors, who were used as the reference category (Table 2).

The estimated cure fraction obtained from the fitted model, which considered the effect of these clinical and pathological characteristics, was 67.0%.

## DISCUSSION

In the current study, factors such as age at diagnosis, BMI, tumor grade, gender, and pathologic stage of the primary tumor were risk factors for CRC. The proportion of cured patients was 64.1% (95% CI, 56.7 to 72.4). The median and mean survival times for the uncured patients were 1,243.83 (95% CI, 1,174.65 to 1,313.00) and 1,493.00 days (95% CI, 1,398.67 to 1,587.33), respectively. The estimated proportion of cured patients (67.0%) was similar to the observed cure fraction (64.1%) obtained from the Kaplan-Meier survival curve. This proportion of cured patients with CRC is slightly higher than the estimate of approximately 60% for Asian populations [22].

BMI, tumor grade, and the pathologic stage of the primary tumor were the 3 main factors associated with survival among patients with CRC in Taleghani Hospital using a non-parametric non-mixture cure model. Gender did not influence the cure fraction in CRC patients, although studies have suggested that women have an advantage in CRC survival [23-27]. This could be due to the relatively small number of patients in the current study compared to the large datasets used in studies that found gender to be a significant factor. Another reason for this advantage may be differences in age at diagnosis [28]. However, in some studies, there were no significant differences in gender, as in our results [29].

Among the oldest group (>65 years), the survival curve did not seem to show a plateau after 2,000 days of follow-up. However, studies have shown that 70% of CRC cases were diagnosed over the age of 65, and that approximately 75% of CRC deaths occurred in people older than 65 years. In our data, this discrepancy with the results of other studies could have been due to the small number of CRC patients who were cured or because of a higher risk of death in this age range [30,31]. Although patients aged 45-65 had a higher survival probability than those who were older than 65, they took a longer time to reach the plateau, indicating an elevated risk of death compared to their younger counterparts. From the statistical perspective, the differences among survival curves in the various age groups of CRC patients were not significant (p=0.27), and the same result was obtained for the impact of age at diagnosis on the cure fraction. Recent studies have indicated that, under specific conditions, age can have a significant impact on the survival of CRC patients. Other studies have shown that for colon cancer, patient age predicted survival, but for rectal cancer, age did not impact survival [32,33].

The current study indicated that BMI had a significant impact on the cure fraction using the non-mixture cure model. Furthermore, the difference across BMI ranges was significant (p<0.001). Being underweight decreased the cure probability of individuals, and being overweight/obese was protective. Studies have shown discordant results regarding the effect of BMI on the survival of CRC patients. A previous study yielded similar results to our work [34], while other studies have reported contradictory results, finding that obesity was significantly related with a lower cure fraction of CRC [35,36]. These discrepancies could be due to differences in the type of patients included in the study (metastatic and non-metastatic) and the relationship of BMI to the time of diagnosis (pre- and post-diagnosis). A study conducted in the US suggested that pre-diagnosis BMI, but not post-diagnosis BMI, was an important predictor of survival among patients with non-metastatic CRC [37].

Though controversial, tumor grade is generally considered to be a stage-independent prognostic factor, and high-grade or poorly differentiated histology is associated with poor patient survival [38,39]. A study in Iran showed that high tumor grade at the time of disease diagnosis was associated with poor survival of CRC patients [40]. In the current study, moderately and poorly differentiated tumors were associated with a significant reduction in survival (p<0.001).

Although CRC is known to be a fatal disease, significant improvements in therapies and early detection of this cancer have led to improvements in survival. The ‘cure’ models have high-lighted the importance of looking at long-term survival as a measure of improvement in survival, and not just focusing on the benchmark of 5-year survival that is usually used when comparing results over time. Using this more appropriate form of survival analysis can help clinicians and researchers to determine potential risk factors that affect the survival of CRC patients who are not susceptible to the occurrence of the event under study. In this study, we were not able to use all pathological, clinical, and biological characteristics simultaneously in the model due to the problem of multicollinearity. In addition, the current data had a large number of missing variables, and using all variables at the same time made the sample size very small, although a large sample size is a desirable property for cure models [41]. It is recommended to conduct further studies to identify the best parametric cure model for these data based on the Akaike information criterion.

## Figures and Tables

**Table 1.** Descriptive analysis of colorectal cancer risk factors and log-rank test for non-cured patients

Factors	Patients, n (%)	Deaths, n (%)	Log-rank test		
			Mean survival time (yr)	SE	p-value
Age at diagnosis (yr)					0.19
<45	146 (28.5)	31 (35.2)	3.42	0.12	
45-65	256 (50.0)	39 (44.3)	3.40	0.14	
>65	110 (21.5)	18 (20.5)	3.41	0.10	
BMI (kg/m^2^)					0.007
≤18.5	46 (9.0)	14 (15.9)	2.64	0.25	
18.6-24.9	266 (51.9)	52 (59.1)	3.28	0.16	
25.0-29.9	157 (30.7)	16 (18.2)	3.67	0.16	
>29.9	43 (8.4)	6 (6.8)	3.66	0.23	
Tumor grade					<0.001
Well	289 (56.4)	34 (38.6)	3.63	0.11	
Moderately	177 (34.6)	38 (43.2)	3.19	0.17	
Poorly	46 (9.0)	16 (18.2)	2.73	0.29	
Gender					0.82
Men	309 (60.3)	55 (62.5)	3.42	0.13	
Women	203 (39.6)	33 (37.5)	3.40	0.14	
Pathologic stage of the primary tumor					0.15
T0	439 (85.7)	63 (71.6)	3.40	0.09	
T1	73 (14.3)	25 (28.4)	3.22	0.21	

SE, standard error; BMI, body mass index.

**Table 2.** Promotion time cure model results, univariate analysis, and multivariate analysis of the survival of colorectal cancer patients using the cure fraction

Factors	Univariate		Multivariate	
	Estimate	p-value	Estimate	p-value
Age at diagnosis (yr)				
<45	Reference		Reference	
45-65	-0.03	0.16	-0.36	0.60
>65	0.01	0.97	0.15	0.62
Body mass index (kg/m^2^)				
≤18.5	Reference		Reference	
18.6-24.9	-0.04	0.16	-0.60	0.04
25.0-29.9	-1.31	<0.001	-1.43	<0.001
>29.9	-0.99	0.04	-0.93	0.06
Tumor grade				
Well	Reference		Reference	
Moderately	0.55	0.02	0.46	0.06
Poorly	1.11	<0.001	1.17	<0.001
Gender				
Men	Reference		Reference	
Women	-0.15	0.50	-0.03	0.90
Pathologic stage of the primary tumor				
T0	Reference		Reference	
T1	0.69	<0.001	0.58	0.01
